# Retraction: Structural characterization of ginseng oligopeptides and anti-aging potency evaluation in *Caenorhabditis elegans*

**DOI:** 10.1039/d2ra90048c

**Published:** 2022-05-04

**Authors:** Laura Fisher

**Affiliations:** Royal Society of Chemistry Thomas Graham House, Science Park, Milton Road Cambridge CB4 0WF UK advances-rsc@rsc.org

## Abstract

Retraction of ‘Structural characterization of ginseng oligopeptides and anti-aging potency evaluation in *Caenorhabditis elegans*’ by Qiang Luo *et al.*, *RSC Adv.*, 2020, **10**, 39485–39494, https://doi.org/10.1039/D0RA06093C.

The Royal Society of Chemistry hereby wholly retracts this *RSC Advances* article due to a significant amount of unattributed text overlap with articles by different author groups that were not cited, including articles published in *Phytochemistry* by Shan Su *et al.*^1^ and *Journal of Functional Foods* by Elena M. Vayndorf *et al.*^2^

Zhigang Liu agrees to the retraction. The other authors have been informed but have not responded to any correspondence regarding the retraction.

Signed: Laura Fisher, Executive Editor, *RSC Advances*

Date: 25^th^ April 2022

## Supplementary Material
